# Repeated assessment of work-related exhaustion: the temporal stability of ratings in the Lund University Checklist for Incipient Exhaustion

**DOI:** 10.1186/s13104-020-05142-x

**Published:** 2020-06-26

**Authors:** Roger Persson, Kai Österberg

**Affiliations:** 1 Department of Psychology, Lund University, Lund, 22100 Sweden; 2 Department of Laboratory Medicine, Division of Occupational and Environmental Medicine, Lund University, Lund, 22185 Sweden

**Keywords:** Big five, Burnout, Exhaustion disorder, LUCIE, Mental health, Personality, Screening, Stress, Trait, Work

## Abstract

**Objective:**

Screening inventories are important tools in clinical settings and research but may be sensitive to temporary fluctuations. Therefore, we revisited data from a longitudinal study with the Lund University Checklist for Incipient Exhaustion (LUCIE) that comprised occupationally active individuals (n = 1355; 27–52 years; 57% women) and one initial paper and pencil survey and 10 subsequent equally spaced online surveys. In the present study we examine to what extent the LUCIE scores changed across 3 years (11 assessments) and whether episodes of temporary elevated LUCIE scores (LTE) coincided with reports of negative or positive changes at work or in private life.

**Results:**

In the total sample, the prevalence rates for the four LUCIE classifications of signs of increasing exhaustion (from no exhaustion to possible exhaustion disorder) ranged from 65.4–73.0%, 16.6–20.9%, 6.2–9.6%, and 3.4–5.0%. Of 732 individuals screened for LTE episodes, 16% had an LTE episode. The LTE episodes typically coincided with reports of adverse changes at work or, to a lesser extent, in private life. Thus, LUCIE classifications appear reliable and lend themselves to repeated use on the same individuals, or group of individuals. Even single episodes of elevated LUCIE scores seem appropriately to indicate adverse reactions to the work situation.

## Introduction

Screening inventories are important tools in occupational health care and research settings. However, for practical and economic reasons, they are typically applied only once and may thus be sensitive to temporary fluctuations related to the individual, the context, or statistical phenomena (e.g., regression to the mean) [1]. During repeated assessment, the complexity of the test, the number of administrations and the time between assessments is also a concern [2, 3]. Because re-test effects can create ambiguous results and contribute to unreliable classifications of various medical and psychiatric conditions, it is essential to understand the temporal stability of test scores [2, 4, 5].

To further the knowledge on repeated assessment of work-related exhaustion, we re-visited a validation study entailing the Lund University Checklist for Incipient Exhaustion (LUCIE) and 11 assessments across 3 years [6–8]. LUCIE is intended to assess behaviors, feelings and symptoms associated with prodromal stages of exhaustion disorder (ED) [6, 7]. As such, it aligns with clinical experience and research that suggest that early detection/intervention is important [9, 10]. The present objective was to examine how stress and exhaustion warning scores changed across the study period and whether episodes of temporary elevations in LUCIE was associated with personality trait scores or coincided with reports of negative or positive changes at work or in private life. Presumably, temporary elevations that coincides with reported changes in work and/or private life would indicate that LUCIE has an appropriate sensitivity to real life changes. The research questions were:

- To what extent is the point prevalence of stress and exhaustion warnings in LUCIE stable across 11 consecutive measurements?
- Are temporary stress or exhaustion warnings commonly occurring and are they preceded, or concurrent, with reports of changes at work and/or in private life?
- Do individuals with temporary elevated stress or exhaustion warnings differ from individual’s never displaying stress or exhaustion warnings, regarding demographic characteristics, personality traits and descriptions of work and private life stressors.

## Main text

### Methods

#### Participants and study design

Occupationally active individuals (n = 1355; 57% women), who had replied to a previous population survey [11] or been randomly drawn from a population registry [6], completed one paper and pencil survey (T0; spring 2012) and 10 equally spaced (i.e., 3 months) online surveys (T1 to T10; September 2012 to December 2014) [6–8]. Their mean age was 41.1 years (SD 6.7 years; range 27 − 52). The main pool of participants is identical to previous study samples [6–8].

#### Measures

LUCIE entails 28 items covering six domains that make up two supplementary scales: the *Stress Warning Scale (SWS)* (0–100) and the *Exhaustion Warning Scale (EWS)* (0–100). Using pre-defined cut-off scores on each scale, the SWS and EWS are combined into a four-step ladder of incremental stress symptomatology: STEP 1-GG (normal: SWS green zone and EWS green zone), STEP2-YG (SWS yellow zone and EWS green zone), STEP 3-RG (SWS red zone and EWS green zone), and STEP 4-RR (possible ED: SWS red zone and EWS red zone). For details on the scoring and development of LUCIE see Persson et al. [7].

Passing episodes of elevated SWS and EWS scores (i.e., LUCIE Temporary Elevation [LTE]) were identified for each individual. An LTE episode/case was defined by temporarily scoring in the red zone on either scale (i.e., *Step 3*-*RG* or *Step 4*-*RR*) while scoring at Step 1-GG or Step 2-YG in the assessment before and after. Given this definition and study design, up to 5 LTE episodes per individual could be achieved.

Personality traits were assessed in five dimensions at T0 with a Swedish 44-item version of the Big Five Inventory (BFI) [12, 13].

Two forced choice items asked: “Has your situation at work (alternatively in your private life) changed in a positive or negative direction during the past couple of months?” [6]. Participants were also encouraged to complete an optional free-text field (480 signs).

#### Data management, statistical analysis and analysis of free-text answers

LTE cases were drawn from the control group sample (n = 745) in a previous study [6]. None of these participants (n = 745) had showed a sustained stress or exhaustion warning (i.e., over several consecutive quarters) in the previous longitudinal study [6] but some, however, displayed intermittent elevations in LUCIE scores (i.e., only one quarter). Thus, we targeted only control group participants with intermittent LTE episodes. In this group, 82% had a completed all 11 surveys, 17% failed to reply to 1 to 3 surveys, and < 1% failed to respond to ≥ 4 surveys [6].

Because the items “*Changes in the situation at work and in private life*” were introduced at T1, the search of LTE cases entailed waves T1 to T10 and 732 individuals. When LUCIE scores across three consecutive quarters (Q) confirmed an LTE for the first time, the elevation phase was set to Q2, the preceding phase to Q1 and the return phase to Q3. The LTE data was compiled into a new data set and merged with the data from non-LTE participants at T8 to T10.

Statistical analysis applied traditional non-parametric and parametric testing using the IBM/SPSS software version 25 (two-tailed alpha level was set to ≤ 0.05). Sensitivity analyses evaluated potential effects of participant dropout. Thematic analyses of free-text commentaries sufficed using the categories established in our previous study [6].

### Results

Both the participation rate and the median SWS scores declined slightly between T0 and T4, but stabilized thereafter (Table 1). Sensitivity analyses entailing the subset of participants that had complete data across the 11 assessments (n = 670; 49%) indicated a similar pattern of decline in SWS scores. The median EWS score exhibited mostly a floor-effect throughout the study (Table 1).

**Table 1:** Distribution of prevalence rates and median scores (Mdn) with accompanying 95% confidence intervals [95% CI] for LUCIE classes (Step 1 GG to Step 4 RR) and the SWS and EWS scales across the 11 assessments rounds for the total study sample at each round

	Step 1 GG			Step 2 YG			Step 3 RG			Step 4 RR^1^			Total (n = 1355)	Participation rate	SWS		EWS	
	N	%	[95% CI]	N	%	[95% CI]	N	%	[95% CI]	N	%	[95% CI]	N	%	Mdn	[95% CI]	Mdn	[95% CI]
T0	881	65.4	[63.0–68.0]	282	20.9	[18.9–23.2]	117	8.7	[7.3–10.3]	67	5.0	[3.9–6.3]	1347	99.4	11.1	[8.7–11.1]	0	[0.0–0.0]
T1	824	69.0	[66.3–71.6]	229	19.2	[17.1–21.5]	84	7.0	[5.7–8.6]	57	4.8	[3.7–6.1]	1194	88.1	8.3	[7.1–9.7]	0	[0.0–0.0]
T2	816	67.4	[64.7–70.0]	222	18.3	[16.3–20.6]	116	9.6	[8.1–11.4]	56	4.6	[3.6–6.0]	1210	89.3	8.3	[5.7–9.7]	0	[0.0–0.0]
T3	827	69.7	[67.0–72.2]	227	19.1	[17.0–21.5]	85	7.2	[5.8–8.8]	48	4.0	[3.1–5.3]	1187	87.6	7.1	[5.6–8.3]	0	[0.0–0.0]
T4	710	70.0	[67.1–72.7]	188	18.5	[16.3–21.0]	78	7.7	[6.2–9.5]	39	3.8	[2.8–5.2]	1015	74.9	5.6	[5.6–8.3]	0	[0.0–0.0]
T5	779	72.2	[69.6–74.8]	184	17.1	[14.9–19.4]	79	7.3	[5.9–9.0]	37	3.4	[2.5–4.7]	1079	79.6	5.6	[5.6–7.1]	0	[0.0–0.0]
T6	739	71.0	[68.1–73.7]	174	16.7	[14.6–19.1]	77	7.4	[6.0–9.2]	51	4.9	[3.8–6.4]	1041	76.8	5.6	[5.6–8.3]	0	[0.0–0.0]
T7	772	72.8	[70.0–75.4]	194	18.3	[16.1–20.7]	56	5.3	[4.1–6.8]	39	3.7	[2.7–5.0]	1061	78.3	5.6	[5.6–5.6]	0	[0.0–0.0]
T8	725	72.3	[69.4–75.0]	175	17.4	[15.2–19.9]	64	6.4	[5.0–8.1]	39	3.9	[2.9–5.3]	1003	74.0	5.6	[5.6–7.1]	0	[0.0–0.0]
T9	739	73.0	[70.1–75.6]	168	16.6	[14.4–19.0]	63	6.2	[4.9–7.9]	43	4.2	[3.2–5.7]	1013	74.7	5.6	[5.6–5.6]	0	[0.0–0.0]
T10	714	71.0	[68.1–73.7]	172	17.1	[14.9–19.6]	75	7.5	[6.0–9.3]	45	4.5	[3.4–5.9]	1006	74.2	5.6	[5.6–7.1]	0	[0.0–0.0]
M(a)	775.1	70.3	[68.0–73.0]	201.4	18.1	[16.0–20.4]	81.3	7.3	[5.8–8.8]	47.4	4.3	[3.1–5.5]	1105.1	81.5	6.7	[5.2–8.2]	–	–
M(h)	771.5	70.3	[67.6–73.0]	196.6	18.0	[15.7–20.3]	77.5	7.1	[5.6–8.6]	45.8	4.2	[3.0–5.4]	1095.4	80.8	6.4	[5.0–7.8]	–	–

Cut-off scores for the SWS score: ≤ 17.00 (‘the green zone’); 17.01 and 38.50 (‘the yellow zone’); ≥ 38.51 (‘the red zone’). Cut-off scores for the EWS score: ≤ 21.50 (‘the EWS green zone’); > 21.50 (‘the EWS red zone’)

*T0* Spring 2012, *T1* September 2012, *T10* December 2014; *M(a)* Arithmetic mean, *M(h)* Harmonic mean, *SWS* Stress Warning Scale, *EWS* Exhaustion Warning Scale

^1^The rare LUCIE combination of SWS yellow + UWS red are included in this category; Step 1-GG (SWS green zone and EWS green zone) = no or negligible lasting stress symptoms. Step 2-YG (SWS yellow zone and EWS green zone) = possible slight lasting stress symptoms. Step 3-RG (SWS red zone and EWS green zone) = mild to moderate lasting stress symptoms, but less severe than Exhaustion Disorder (ED). Step 4-RR (SWS red zone and EWS red zone) = lasting stress symptoms of a severity indicating possible ED. LUCIE covers six dimensions: (a) sleep and recovery, (b) separation between work and spare time, (c) sense of community and support in the workplace, (d) managing work duties and personal capabilities, (e) private life and spare time activities, and (f) health complaints

Across the 11 assessments, the prevalence rates ranged from 3.4% to 5.0% for Step 4-RR, 6.2% to 9.6% for Step 3-RG, 16.6% to 20.9% for Step 2-YG, and 65.4% to 73.0% for Step 1-GG (Table 1; Additional file 1). Spearman rho correlations between the LUCIE steps ranged from 0.41 to 0.64 (p < 0.001), showing a trend of decreasing coefficient values in relation to increasing time between measurements (Additional file 2).

The analysis of LTE episodes showed that 16% (n = 116) exhibited an LTE (T2: n = 23, T3: n = 13, T4: n = 14, T5: n = 11, T6: n = 25, T7: n = 9, T8: n = 12, and T9: n = 9) whereas 616 did not (here after denoted the control group). While 89 had one LTE, 22 had two LTE’s, and 5 participants had three LTE’s. Most demographic variables were similar among LTE cases and controls. However, among LTE cases the proportion of women were higher than among controls (χ^2^: p = 0.003) (Table 2).

**Table 2:** Baseline demographical characteristics and personality traits according to the Big Five Personality Inventory (BFI) of the participants identified as having a LUCIE temporary elevation (LTE) and participants without any LTE across the 11 assessments (controls)

Characteristic	LTE (n = 116)	2 or more LTE indications (n = 27)	Controls (n = 616)	LTE (n = 116) versus Controls (n = 616)
				*P* value
Age				0.23
Mean (SD)	41.9 (6.4)	41.1 (7.5)	41.1 (6.5)	
Range	27–52	27–52	27–52	
Gender (%)				0.003
Men	34	22	51	
Women	66	78	49	
Education (%)				0.059
Nine-year compulsory schooling	1	0	0	
Upper secondary school	23	22	26	
University studies	76	78	74	
Occupational activity (%)				0.78
Full-time work (≥ 40 h/week)	83	81	81	
Part-time work (30–39 h/week)	16	19	18	
Part-time work (20–29 h/week)	1	0	1	
Employment (%)				0.46
Salaried employee	89	93	92	
Self-employed	4	0	5	
Combined self-employment and employee	6	7	4	
BFI personality dimension	M (SD)	–	M (SD)	*P* value $\eta_{p}^{2}$
Neuroticism	2.58 (.61)	–	2.29 (.57)	<0.001 (0.034)
Extraversion	3.59 (.65)	–	3.59 (.67)	0.96
Openness	3.47 (.62)	–	3.39 (.60)	0.19
Agreeableness	3.87 (.42)	–	3.95 (.44)	0.076
Conscientiousness	3.91 (.46)	–	3.95 (.49)	0.37

An LTE episode/case was defined by temporarily scoring in the red zone on the LUCIE SWS or EWS scales (i.e., Step 3-RG or Step 4-RR) while scoring at Step 1-GG or Step 2-YG in the assessment before and after. Comparisons with categorical data were made with Pearson Chi Square tests. Comparisons involving continuous outcomes were made with one-way analysis of variance F-tests (ANOVA)

*LTE* LUCIE temporary elevation

The SWS and EWS scores were generally higher in the LTE group than in the control group across all three quarters (p < 0.001; Mann–Whitney U-test; Additional file 3), and most clearly so at Q2 (Elevation phase).

**Fig. 1:**
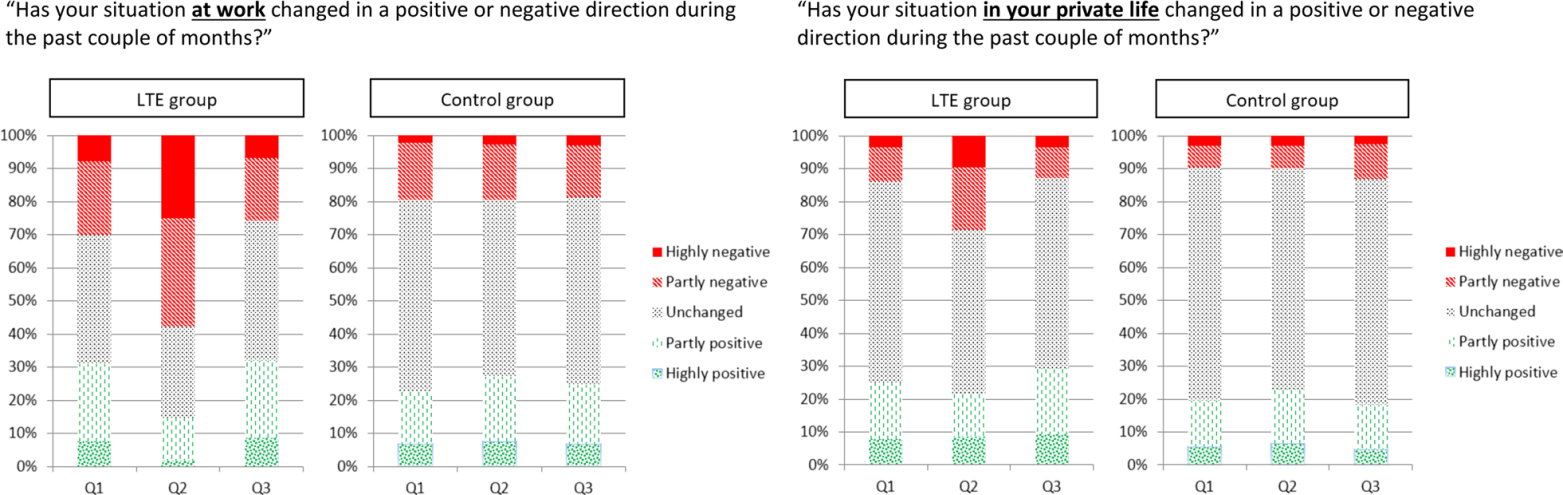
Ratings of changes in the work situation (left graph) and in the private situation (right graph). Within each graph the left panel shows ratings during the three quarters of fulfillment of the criterion among LUCIE temporary elevated cases (LTE; n = 116), whereas the right panel shows the corresponding data for controls (n = 616)

Ratings of both negative and positive *changes at work* were more frequent among LTE cases (71% and 54%, respectively) than among controls (39% and 46%, respectively) (χ^2^: p < 0.001; Fig. 1; Additional file 4). For both type of ratings, the largest difference occurred at Q2, at which 19% among controls, and 58% among LTE cases, reported a partly or highly negative change at work (χ^2^: p < 0.001). Contrariwise, 27% of the controls reported a partly or highly positive change at work whereas only 15% of the LTE cases did (χ^2^: p < 0.001).

Ratings of negative and positive *changes in the private life* were more frequent among LTE cases (41% and 49%, respectively) than among controls (23% and 38%, respectively) (χ^2^: p < 0.001; Fig. 1; Additional file 4). For ratings of negative changes, the largest difference occurred at Q2, at which 10% among controls and 28% of LTE cases reported a partly or highly negative changes in their private situation (χ^2^: p < 0.001). For ratings of positive changes, the largest difference occurred at Q3, at which 18% among controls and 29% of the LTE cases reported a higher rate of positive changes in the private situation (χ^2^: p = 0.006).

The analysis of the free-text commentaries gave a deeper understanding of complaints, and delineated the interplay between work life and private life. See Additional files 5 and 6 for a listing and in depth analysis of free-text answers, respectively. Noticeably, however, when analyzing the 45 free-text answers from the in total 48 LTE cases that had rated negative changes in private life on the forced choice item, it became clear that some had misattributed a negative impact from work as a “negative change in private life”. Thus, if discounting reports like “feeling worn out due to work” and reports flagging spillover from work to family as a private burden, only 29% had a solely (genuine) private burden *unrelated* to work in the total group of 116 participants with an LTE, in contrast to the 41% reported above (see Additional file 6 for computation details).

Reports of *simultaneous* negative changes at work and in the private sphere were infrequent among LTE cases at Q1(7%) and Q3(3%) but rose to 20% at Q2. Some 20% of LTE cases did not report any negative change at work or in the private sphere during Q1 to Q3, see Additional file 7 for further details.

LTE cases had higher Neuroticism scores than controls (ANOVA p < 0.001; $\eta_{p}^{2}$ = 0.034) (Table 2).

### Discussion

The prevalence rates for the stress and exhaustion warnings in LUCIE (i.e., Step 1-GG to Step 4-RR) were essentially stable throughout the study period, although the median SWS scores declined between T0 and T4 indicating a weak drift towards better health. Conspicuously, the participation rates declined in parallel. However, the sensitivity analyses rejects participant dropout as an explanation for the decreasing SWS scores.

Noticeably, only 16% displayed an LTE, and women were overrepresented with a ratio of 2:1. Despite a minute effect size, the higher neuroticism scores among LTE cases corroborates previous cross-sectional and longitudinal findings suggesting that personality traits and stress reactions to some extent are related [6, 7, 14]. More importantly, however, is that the LTE episodes coincided more frequently with ratings of changes in the work situation, and predominantly so during the elevation phase (Q2), when compared with changes reported to occur in the private life sphere. The analysis of the free-text commentaries strengthened this view. Indeed, some LTE cases misattributed work exposures as being private life stressors. Thus, even a short-term impoverishment of the work situation appears to be associated with the reporting of stress and exhaustion symptoms in LUCIE. In accordance with previous findings in cases of long-term elevation of LUCIE-scores [6], LUCIE appear to be a sensitive measure of short-term stress symptoms/signs related to primarily the work situation and, as such, is probably a useful tool in the clinical screening of early signs of stress symptomatology and exhaustion in working populations.

Although LTE cases more frequently reported both negative and positive changes at work and, to a lesser extent, in the private situation, 20% of the LTE cases did not report any negative change whatsoever. This puzzle remains even after analyzing the LTE episodes in relation to a control question, documenting the occurrences of circumstances that in theory could have biased the replies in the original survey (e.g., pregnancy, menopause, pain, somatic disease, disturbed sleep due to small children or late habits, or other unspecified private life burdens; data not shown). Yet, humans sometimes display symptoms without being able to attribute them to a specific external or internal factor. Such unknown, or random, variation underlines that results from screening instruments on the individual level is only fully understood in a confident dialogue with the person screened. Since temporary fluctuations in mood and performance may occur even in the absence of any identifiable factor known to the individual, single temporary elevations in LUCIE scores should be conceived as *possible* indications of increased stress symptoms.

### Conclusions

Participation rates and median stress warning scores declined independently from each other during the first five assessments rounds but stabilized thereafter. The overall pattern of results suggest that LUCIE classifications are reliable and lend themselves to repeated use on the same individuals, or group of individuals. Thus, even single episodes of elevated LUCIE scores seem appropriately to indicate adverse reactions to the work situation.

## Limitations

Since the participants had long education and all were healthy when entering the study, the results may underestimate population levels of stress and exhaustion warnings and the occurrence of temporary elevations (LTE episodes). The calculations of 95% confidence intervals (CI), and analysis of LTE data, did not account for clustering within individuals. Thus, the CI’s may be too narrow due to an underestimation of the standard errors.

## Supplementary information

**Additional file 1:** Graphical overview of the point prevalence rates in LUCIE across the 11 consecutive assessments.

**Additional file 2:** Spearman rho correlation coefficients for the four-step severity ladder of stress symptomatology across the 11 quarters of the study period.

**Additional file 3:** Stress Warning and Exhaustion Warning scores during LTE episodes and among controls.

**Additional file 4:** Overview and comments on the participants reports of negative and positive changes in the work situation and in the private life sphere.

**Additional file 5:** Frequencies of main themes of positive/negative changes in the work situation and private life during LTE episodes.

**Additional file 6:** Description of the qualitative thematic analysis of the responses to the optional free-text commentaries among LTE cases.

**Additional file 7:** Overview of the analyses of simultaneous descriptions of changes at work and in the private life sphere among LTE cases.

## Data Availability

Consistent with the study protocol approved by the Regional Ethical Review Board, anonymized data is stored locally at the Division of Occupational and Environmental Medicine, Lund University, Lund, Sweden. Because the participants (in accordance with the approved study protocol) were guaranteed that the crude data should not be published on the internet, access to data will only be granted to eligible researchers wanting to audit our research.

## Abbreviations

CI: Confidence interval
LUCIE: Lund University Checklist for Incipient Exhaustion
ED: Exhaustion disorder
SWS: Stress warning scale
EWS: Exhaustion warning scale
Step 1-GG: SWS Green zone and EWS Green zone
Step 2-YG: SWS Yellow zone and EWS Green zone
Step 3-RG: SWS Red zone and EWS Green zone
Step 4-RR: SWS Red zone and EWS Red zone
LTE: LUCIE temporary elevation
Q1: First quarter–preceding phase
Q2: Second quarter–elevation phase (LTE)
Q3: Third quarter–return phase
