# Supplementary material for: Repeated assessment of work-related exhaustion: the temporal stability of ratings in the Lund University Checklist for Incipient Exhaustion
Source: BMC Res Notes. 2020 Jun 26;13:304. doi: 10.1186/s13104-020-05142-x (PMC7318754; doi:10.1186/s13104-020-05142-x)
Supplement: Supplementary file 1 — Additional file 1: Graphical overview of the point prevalence rates in LUCIE across the 11 consecutive assessments. [file 13104_2020_5142_MOESM1_ESM.docx]

**Additional file 1**

This file shows a graphical overview of the point prevalence rates in LUCIE across the 11 consecutive assessments (n = 1347 [T0] to 1003 [T8]).

**Figure 1:1**

Figure 1:1 Proportions of LUCIE classes (prevalence rates) across the 11 assessments rounds for the total study sample at each round. T0 = Spring 2012, T1= September 2012, T10 =December 2014.

- Step 1-GG: SWS green zone and EWS green zone = no or negligible lasting stress symptoms.
- Step 2-YG: SWS yellow zone and EWS green zone = possible slight lasting stress symptoms.
- Step 3-RG: SWS red zone and EWS green zone = mild to moderate lasting stress symptoms, but less severe than meeting criteria for Exhaustion Disorder.
- Step 4-RR: SWS red zone and EWS red zone = lasting stress symptoms of a severity indicating possible Exhaustion Disorder.
