# Supplementary material for: Repeated assessment of work-related exhaustion: the temporal stability of ratings in the Lund University Checklist for Incipient Exhaustion
Source: BMC Res Notes. 2020 Jun 26;13:304. doi: 10.1186/s13104-020-05142-x (PMC7318754; doi:10.1186/s13104-020-05142-x)
Supplement: Supplementary file 2 — Additional file 2: Spearman rho correlation coefficients for the four-step severity ladder of stress symptomatology across the 11 quarters of the study period. [file 13104_2020_5142_MOESM2_ESM.docx]

**Additional file 2**

Table 2:1 Spearman rho correlation coefficients for the four-step severity ladder of stress symptomatology (Step 1-GG, Step 2-YG, Step 3-RG and Step 4-RR) across the 11 quarters of the study period. Sample sizes ranged from *n*=876 to 1202.

|  | **T0** | **T1** | **T2** | **T3** | **T4** | **T5** | **T6** | **T7** | **T8** | **T9** | **T10** |
| --- | --- | --- | --- | --- | --- | --- | --- | --- | --- | --- | --- |
| **T0** | ... |  |  |  |  |  |  |  |  |  |  |
| **T1** | .60 | .. |  |  |  |  |  |  |  |  |  |
| **T2** | .55 | .64 | .. |  |  |  |  |  |  |  |  |
| **T3** | .54 | .55 | .59 | .. |  |  |  |  |  |  |  |
| **T4** | .52 | .50 | .57 | .59 | .. |  |  |  |  |  |  |
| **T5** | .51 | .50 | .48 | .51 | .59 | .. |  |  |  |  |  |
| **T6** | .48 | .43 | .47 | .47 | .55 | .57 | .. |  |  |  |  |
| **T7** | .44 | .41 | .49 | .45 | .52 | .51 | .60 | .. |  |  |  |
| **T8** | .42 | .42 | .45 | .46 | .48 | .48 | .53 | .57 | .. |  |  |
| **T9** | .44 | .44 | .52 | .45 | .50 | .50 | .52 | .52 | .54 | .. |  |
| **T10** | .45 | .44 | .48 | .45 | .51 | .42 | .51 | .51 | .52 | .57 | .. |

Note. All correlation coefficients shown above had p-values < 0.001.

Step 1-GG (SWS green zone and EWS green zone) = no or negligible lasting stress symptoms. Step 2-YG (SWS yellow zone and EWS green zone) = possible slight lasting stress symptoms. Step 3-RG (SWS red zone and EWS green zone) = mild to moderate lasting stress symptoms, but less severe than ED.

Step 4-RR (SWS red zone and EWS red zone) = lasting stress symptoms of a severity indicating possible ED.
