# Supplementary material for: Repeated assessment of work-related exhaustion: the temporal stability of ratings in the Lund University Checklist for Incipient Exhaustion
Source: BMC Res Notes. 2020 Jun 26;13:304. doi: 10.1186/s13104-020-05142-x (PMC7318754; doi:10.1186/s13104-020-05142-x)
Supplement: Supplementary file 3 — Additional file 3: Stress Warning and Exhaustion Warning scores during LTE episodes and among controls. [file 13104_2020_5142_MOESM3_ESM.docx]

**Additional file 3**

Figure 3:1 Stress Warning and Exhaustion Warning scores. A comparison between the participants LTE episodes (n=116) and controls (N=616) in LUCIE.


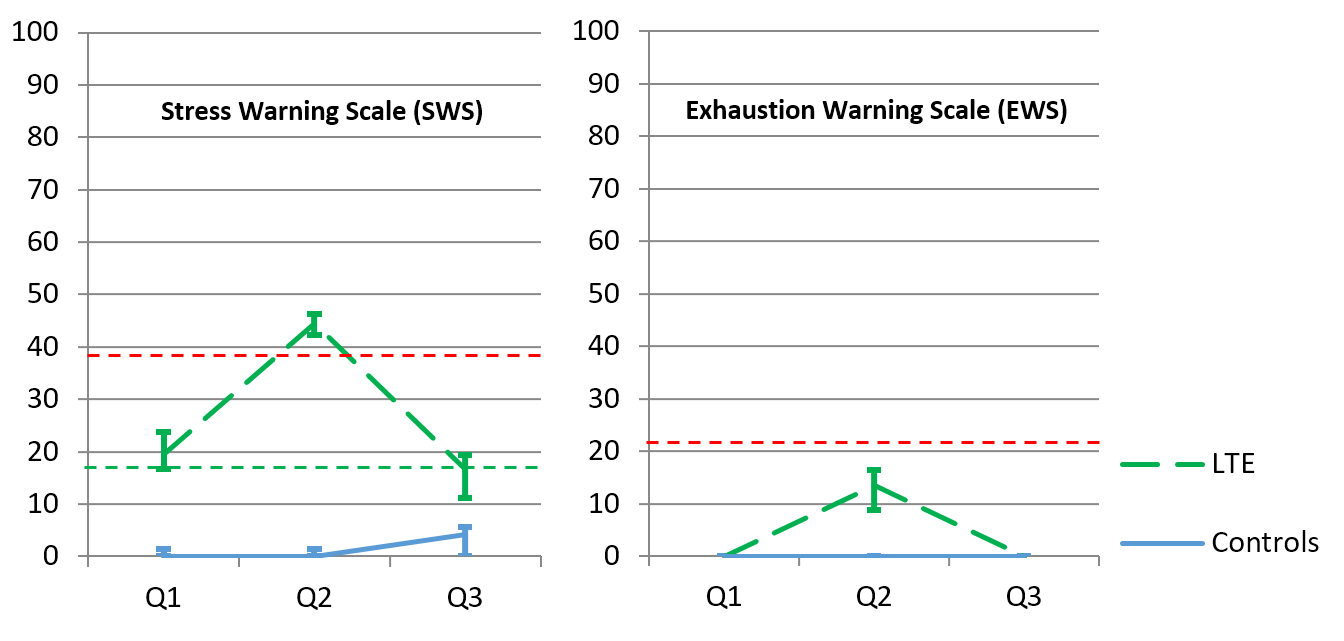


Figure 3:1. LUCIE median values (95% CI) during three quarters. Left panel show SWS values for the subgroup with LUCIE temporary elevation (LTE; n=116) and controls (n=616). Right panel show EWS values for the same groups. The thin horizontal lines (broken) show the borders between SWS-green/yellow/red zones (left panel) and between EWS-green/red zones (right panel).

**Comment on median SWS and EWS values in the preceding phase (Q1), elevation phase (Q2) and the return phase (Q3).**

In total, 89 individuals had one LUCIE Temporary Elevation (LTE) episode, 22 individuals had two LTE episodes, and 5 individuals had three LTE episodes. The data analysis focused on examining the individuals *first* LTE episode (n=116).

Among LTE cases, the median SWS score during the preceding phase at Q1 was 19.4, rising to 44.4 during the elevation phase at Q2, and falling to 16.7 during the return phase at Q3. In contrast, the controls had median SWS scores between 0 (zero) and 4.2. Regarding the EWS scores, the median EWS values were (zero) at Q1 and Q3 among both LTE subjects and controls. However, during the elevation phase at Q2 the EWS scores were higher in the LTE group than among controls (13.5 vs. 0).

Thereafter, the SWS and EWS scores in the LTE group (N=116) were combined into the four steps of increasing exhaustion symptoms (i.e., Step 1-GG, Step 2-YG, Step 3-RG and Step 4-RR) (n=116). When doing this we observed that 43% of the LTE cases had a *Step 1-GG-*result in the preceding phase at Q1 whereas 57 % had a *Step 2-YG-*result. Next and, during the elevation phase at Q2, we observed that 71 % of the LTE cases had a *Step 3-RG*–result whereas 29 % had a *Step 4-RR*-result. Finally, and in the return phase at Q3, we observed that 55 % of the LTE cases had a *Step 1-GG-*result whereas 45 % had a *Step 2-YG*-result.
