# Supplementary material for: Repeated assessment of work-related exhaustion: the temporal stability of ratings in the Lund University Checklist for Incipient Exhaustion
Source: BMC Res Notes. 2020 Jun 26;13:304. doi: 10.1186/s13104-020-05142-x (PMC7318754; doi:10.1186/s13104-020-05142-x)
Supplement: Supplementary file 4 — Additional file 4: Overview and comments on the participants reports of negative and positive changes in the work situation and in the private life sphere. [file 13104_2020_5142_MOESM4_ESM.docx]

**Additional file 4**

This file gives an overview and comments on the participants reports of negative and positive changes in the work situation and in the private life sphere. The figures in this file are the same as Figure 1 in the research note.

**Figure 4:1**


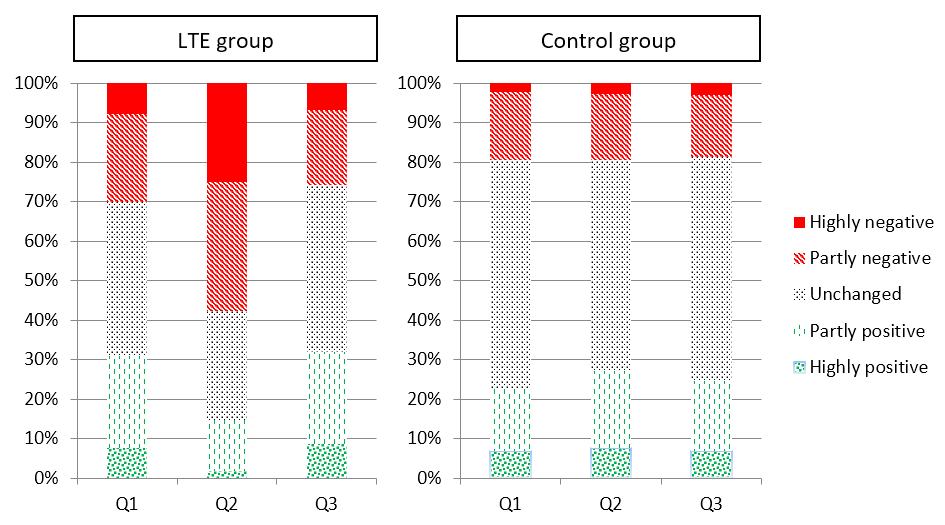


Figure 4:1. Ratings of changes in the work situation. Left panel shows ratings during the three quarters of fulfillment of the criterion among LUCIE temporary elevated cases (LTE; n=116), and right panel shows the corresponding data for controls (n=616).

**Comment to figure 4:1 concerning reported changes in the work situation**

Of the 116 participants with an LUCIE Temporary Elevation (LTE), 71 % reported at least at one point in time *negative* changes in the work situation, as compared with 39 % of the controls (Pearson’s χ2-test: p<0.001). While the 616 controls exhibited a stable level throughout, a larger proportion of participants with an LTE reported a partly or highly negative change at work at Q2 (19 % Vs. 58 %, respectively; ꭓ2: p<0.001). At Q1 and Q3, 30 % and 26 % in LTE group, respectively, gave negative ratings whereas 19 % of the controls did (Q1: ꭓ2: p=0.009; and Q2: ꭓ2: p=0.075).

Of the 116 participants in the LTE group 54 % rated a *positive* change in the work situation at one point, as compared with 46% of the controls (ꭓ2: p<0.069). Only at Q2, did the LTE group differ from controls as 15% reported a partly or highly positive change at work, compared to 27% among controls (ꭓ2: p<0.001). The corresponding figures for Q1 and Q3 was Q1 = 31% and 23%, respectively; p=0.055; and Q3 = 32% and 25%, respectively; ꭓ2: p=0.10.

**Figure 4:2**


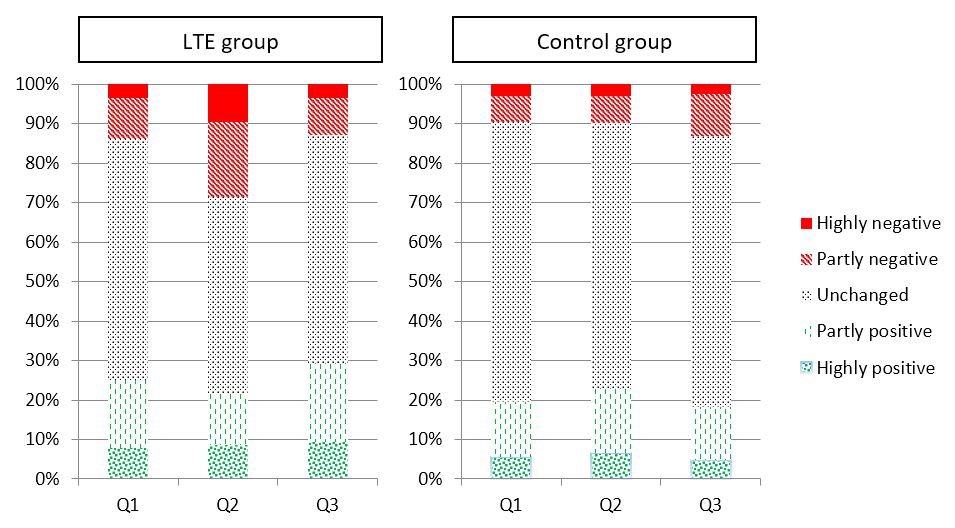


Figure 4:2. Ratings of changes in the private life situation. Left panel shows ratings during the three quarters of fulfillment of the criterion among LUCIE temporary elevated cases (LTE; n=116), and right panel shows the corresponding data for controls (n=616).

**Comment to figure 4:2 concerning reported changes in the private sphere**

In total, 41 % of the 116 participants in the LTE group rated a negative change at least once across the three quarters, compared to 23% among controls (ꭓ2:p<0.001). Especially at Q2, a quite larger proportion of the LTE participants reported having experienced partly or highly negative change in their private situation, that is, 28% compared to 10% among the controls (ꭓ2: p<0.001;) (Figure 4). At Q1 and Q3, LTE participants reported negative changes in the private situation more on a par with controls (Q1: 14% vs. 10%; ꭓ2: p=0.17; Q3: 13% in both groups; ꭓ2: p=0.9).

In total, 49 % of the 116 participants in the LTE group rated a positive change at least once across the three quarters, compared to 38% among controls (ꭓ2: p<0.032) (Figure 4). There proportion of participants that rated positive changes in the private situation did not differ between LTE participants and controls at Q1 and Q2 (Q1 = 25% and 19%, respectively; ꭓ2: p=0.18; Q2 = 22% and 23%, respectively; ꭓ2: p=0.7). However, at Q3 (i.e., the return phase) the LTE group showed a higher rate of positive changes in the private situation than did controls; (29% vs. 18%, respectively; ꭓ2: p=0.006).
