# Supplementary material for: Repeated assessment of work-related exhaustion: the temporal stability of ratings in the Lund University Checklist for Incipient Exhaustion
Source: BMC Res Notes. 2020 Jun 26;13:304. doi: 10.1186/s13104-020-05142-x (PMC7318754; doi:10.1186/s13104-020-05142-x)
Supplement: Supplementary file 5 — Additional file 5: Frequencies of main themes of positive/negative changes in the work situation and private life during LTE episodes. [file 13104_2020_5142_MOESM5_ESM.docx]

**Additional file 5**

This file describes the frequencies of main themes of positive/negative changes in the work situation and private life, which were derived from free-text commentaries among LUCIE Temporary Elevation (LTE) cases (N=116).

Table 5:1. Frequencies of main themes of *negative* changes in the work situation derived from free-text commentaries among participants targeted by the LUCIE LTE algorithm (n=77).

| **Main theme** | **n** | **%** |
| --- | --- | --- |
| Increased workload and/or increased emotional or intellectual demands from employer, including shortage of staff | 40 | 51.3 |
| Organizational problems or negative organizational change, including conflicts within management | 18 | 23.1 |
| Reduced support from supervisor or colleagues | 17 | 21.8 |
| “Job Stress” | 10 | 12.8 |
| Job insecurity | 8 | 10.3 |
| Reduced personal work engagement | 6 | 7.7 |
| Reduced decision latitude or quality of work contents | 5 | 6.4 |
| Injustice on the part of supervisors, including unfairness in work task assignment | 4 | 5.1 |
| [*Unclassifiable comment*] | 3 | 3.8 |
| Less comfortable (or longer) working hours | 2 | 2.6 |
| Reduced reward (salary, acknowledgment) | 2 | 2.6 |
| Multiple worksites/multiple roles | 2 | 2.6 |
| Conflicts with colleagues or supervisors | 1 | 1.3 |

Note. The rates above show the number of individuals reporting a change within each category separately. Thus, any one individual could report changes across several categories.

Table 5:2. Frequencies of main themes of *positive* changes in the work situation derived from free-text commentaries among participants targeted by the LUCIE LTE algorithm (n=59).

| **Main theme** | **n** | **%** |
| --- | --- | --- |
| Improved support from supervisor, colleagues or through group intervention | 13 | 22.0 |
| Reduced workload and/or decreasing emotional or intellectual demands from supervisors, including reduced shortage of staff | 12 | 20.3 |
| Enriched decision latitude or more exciting/stimulating work tasks | 10 | 16.9 |
| Increased reward (salary, acknowledgment, education) | 10 | 16.9 |
| Successful move to new employer (including new self-employment) | 9 | 15.3 |
| [*Unclassifiable comment*] | 6 | 10.2 |
| Reduced stress | 5 | 8.5 |
| Successful organizational change | 2 | 3.4 |
| Improved work space | 2 | 3.4 |
| Improved working hours | 1 | 1.7 |
| Less job insecurity | 1 | 1.7 |
| Education in stress management techniques | 1 | 1.7 |

Note. The rates above show the number of individuals reporting a change within each category separately. Thus, any one individual could report changes across several categories.

Table 5:3. Frequencies of main themes of *negative* changes in the private life situation derived from free-text commentaries among participants targeted by the LUCIE LTE algorithm (n=45).

| **Main theme** | **n** | **%** |
| --- | --- | --- |
| Work-family conflict (lack of time/energy) | 13 | 28.9 |
| Serious illness/death of close relative/close friend | 12 | 26.7 |
| Worn out (fatigue, exhaustion) | 7 | 15.6 |
| Relational problems in family | 6 | 13.3 |
| Worries for children | 6 | 13.3 |
| Negative divorce/separation | 4 | 8.9 |
| Economic stress | 3 | 6.7 |
| Other specific burden | 3 | 6.7 |
| Being fired or spouse fired | 2 | 4.4 |
| Neglected lifestyle/physical fitness | 1 | 2.2 |
| [Unclassifiable comment] | 1 | 2.2 |

Note. The rates above show the number of individuals reporting a change within each category separately. Thus, any one individual could report changes across several categories.

Table 5:4. Frequencies of main themes of *positive* changes in the private life situation derived from free-text commentaries among participants targeted by the LUCIE LTE algorithm (n=51).

| **Main theme** | **n** | **%** |
| --- | --- | --- |
| Improved family relations or family situation | 20 | 39.2 |
| Parenthood/pregnancy (incl. grandchildren) | 11 | 21.6 |
| Improved energy/vitality | 5 | 9.8 |
| Falling in love/new relation | 4 | 7.9 |
| Restructured life plans/attitudes | 4 | 7.9 |
| Voluntary reduction in work hours to cope with life/spending more time with family | 3 | 5.9 |
| Positive divorce/separation | 3 | 5.9 |
| Improved dwellings | 3 | 5.9 |
| Other unspecific improvement | 3 | 5.9 |
| Relaxing vacation | 2 | 3.9 |
| New hobby or new friends | 2 | 3.9 |
| Shorter commuting | 2 | 3.9 |
| Improved lifestyle | 1 | 2.0 |

Note. The rates above show the number of individuals reporting a change within each category separately. Thus, any one individual could report changes across several categories.
