# Supplementary material for: Repeated assessment of work-related exhaustion: the temporal stability of ratings in the Lund University Checklist for Incipient Exhaustion
Source: BMC Res Notes. 2020 Jun 26;13:304. doi: 10.1186/s13104-020-05142-x (PMC7318754; doi:10.1186/s13104-020-05142-x)
Supplement: Supplementary file 6 — Additional file 6: Description of the qualitative thematic analysis of the responses to the optional free-text commentaries among LTE cases. [file 13104_2020_5142_MOESM6_ESM.docx]

# Additional file 6

## This file reports on the qualitative thematic analysis of the LUCIE Temporary Elevation (LTE) cases responses to the optional free-text commentaries (480 signs) to the two force-choice questions: *“Has your situation at work (alternatively in your private life) changed in a positive or negative direction during the past couple of months?”.* This file also supplements “Additional file 5”, which lists the frequencies of the main themes that the thematic analysis revealed, as well as comment on the process of disentangling work-related burdens from private circumstances in the free-text statements.

##

## The Situation at Work

## 6.1 Analysis of free-text answers related to negative and positive changes at work

Among the 116 LUCIE Temporary Elevation (LTE) cases, 82 participants reported a negative change in their work situation and 77 gave a free-text description. The qualitative thematic analysis allowed 12 classifiable categories of complaints (Additional file 5; Table 5:1). The most common category was *increased workload/demands, including shortage of staff* (51%), followed by various *organizational problems or a negative organizational change* (23%), and *reduced support from supervisor or colleagues* (22%).

## 6.2 Analysis of free-text answers related to negative and positive changes at work

Among the 116 LTE cases, 63 participants reported having experienced a positive change at work. Of these, 59 participants gave free-text descriptions allowing 11 classifiable categories (Additional file 5; Table 5:2). The most common categories were *Improved support from supervisor, colleagues or through group intervention* (22%), *Reduced workload and/or decreased emotional or intellectual demands from supervisors, including reduced shortage of staff* (20%), *Enriched decision latitude or more exciting/stimulating work tasks* (17%) and *Increased reward (salary, acknowledgment, education)* (n=17%). In addition, *Successful move to new employer (including new self-employment)* were reported by 15%.

**The Private Life Sphere**

## 6.3 Analysis of free-text answers related to negative changes in the private sphere

Of the 48 participants with an LTE who had rated any negative change in private life during Q1 to Q3, 45 provided free-text descriptions allowing for 10 classifiable categories (see Additional file 5; Table 5:3). The most common categories were *work-family conflict (lack of time/energy)* (N=13; 29%) and *serious illness/death of close relative/close friend* (N=12; 27%). Other common categories were *feeling worn-out (fatigue, exhaustion)* (16%) and *relational problems in family* and *Worries for children* (both 13%).

## 6.4 Analysis of free-text answers related to positive changes in the private sphere

Of the 57 participants with an LTE whom reported a positive change in private life during Q1 to Q3, 51 provided free-text descriptions allowing for 13 classifiable categories (Additional file 5; Table 5:4). *Improved family relations or family situation* was by far the most commonly reported positive change (n=20; 39%), but 22% (n=11) reported *parenthood/pregnancy (including caring for grandchildren)* as being a positive change.

**The Interaction between Work and Private Life**

## 6.5 Disentangling work-related burden from private circumstances in the free-text statements

Observably, in the free-text answers, participants with an LTE indication sometimes described a negative impact from work as a “negative change in private life”. Thus, if discounting descriptions signaling *work-to-family conflict (lack of time/energy)* from “classical” private burdens (i.e., relational problems in the family, death of relatives, problems with children, divorce, etc.), 38 of the 45 respondents reported a negative change. If further discounting descriptions signaling *feelings of being worn-out due to work (fatigue, exhaustion)* from private burdens, 32 of the 45 respondents reported a negative change in their private situation. If extrapolating from the proportion of LTE individuals with “genuine” private life burdens (i.e., 32/45 = 0.71) to represent all 48 negative raters (0.71*48=34), the estimate is that 29% (34/116=29.3%) had a solely (genuine) private burden *unrelated* to work in the total group of 116 participants with an LTE.
