# Supplementary material for: Repeated assessment of work-related exhaustion: the temporal stability of ratings in the Lund University Checklist for Incipient Exhaustion
Source: BMC Res Notes. 2020 Jun 26;13:304. doi: 10.1186/s13104-020-05142-x (PMC7318754; doi:10.1186/s13104-020-05142-x)
Supplement: Supplementary file 7 — Additional file 7: Overview of the analyses of simultaneous descriptions of changes at work and in the private life sphere among LTE cases. [file 13104_2020_5142_MOESM7_ESM.docx]

# Additional file 7

## This file comment on the simultaneous descriptions of changes at work and in the private life that were made by individuals with a LUCIE Temporary Elevation (LTE). As such, the comments concerns the optional free-text commentaries (480 signs) as well as the two questions with forced choice response alternatives, that is, “*Has your situation at work (alternatively in your private life) changed in a positive or negative direction during the past couple of months?”*

## 7:1 Simultaneous descriptions of changes at work and in the private circumstances in the force choice items and the free-text statements

Among the 116 LUCIE Temporary Elevation Cases (LTE), simultaneous reports of negative changes the work situation and in the private sphere were infrequent in the preceding phase at Q1 and in the return phase at Q3 (7 % and 3%, respectively). However, during the elevation phase at Q2, the frequency of simultaneous reports rose to 20%. Noticeably, this seems primarily to have been caused by the rising rate of negative changes in the work situation (from 30% at Q1 to 58% at Q2) as traditional private life stressors increased less (from 14% at Q1 to 28% at Q2). If discounting the inaccurately attributed private burdens that rather reflected negative changes at work (i.e., *work-to-family conflict* and *feeling worn-out due to work),* 12% of the participants in the LTE group exhibited simultaneous ratings of negative changes in the work situation and in the private sphere at Q2.

The proportion of individuals in the LTE group that exclusively reported negative changes in the private sphere (i.e. without reporting a simultaneous negative change in the work situation) was 7 % in the preceding phase at Q1 and 9 % in the elevation phase at Q2 and in the return phase at Q3. If, as described in the previous paragraph, discounting the themes *work-to-family conflict* and *feeling worn-out due to work* the prevalence rates were 6%, 8% and 9%, respectively.

The proportion of individuals in the LTE group that exclusively reported negative changes in the work situation (i.e. without reporting a simultaneous negative change within the private sphere) were in the preceding phase at Q1 22 % and in the return phase at Q3 23 %. During the elevation phase at Q2 38% of the individuals in the LTE group exclusively reported a negative change in the work situation. After excluding the private sphere themes *work-to-family conflict* and *feeling worn-out due to work* – which recalculation made the rates for parallel work-related burden and private burdens drop - the final rates for pure work-related negative changes rose to 46% in quarter 2, while remaining at roughly the same figures as before in the first and middle quarters (23-25%).

 Interestingly, 20% of LTE cases did not report any negative change whatsoever, in either the work situation or the private sphere, during the three quarters.
